# One‐year outcomes for congenital diaphragmatic hernia

**DOI:** 10.1002/bjs5.50135

**Published:** 2019-01-31

**Authors:** Y. Wang, K. Honeyford, P. Aylin, A. Bottle, S. Giuliani

**Affiliations:** ^1^ Dr Foster Unit, Department of Primary Care and Public Health, Imperial College London London UK; ^2^ Department of Specialist Neonatal and Paediatric Surgery Great Ormond Street Hospital for Children NHS Foundation Trust London UK

## Abstract

**Background:**

Congenital diaphragmatic hernia (CDH) is a congenital anomaly with high mortality and long‐term morbidity. The aim of this study was to benchmark trends in 1‐year and hospital volume outcomes for this condition.

**Methods:**

This study included all infants born with CDH in England between 2003 and 2016. This was a retrospective analysis of the Hospital Episode Statistics database. The main outcomes were: 1‐year mortality, neonatal length of hospital stay (nLOS), total bed‐days at 1 year and readmission rate. The association between hospital volume and outcomes was assessed for specialist paediatric surgery centres.

**Results:**

A total of 2336 infants were included (incidence 2·5 per 10 000 live births). No significant time trends were found in incidence and main outcomes. Some 1491 infants (63·8 per cent) underwent surgical repair. The 1‐year mortality rate was 31·2 per cent. Median nLOS and total bed‐days were 17 and 19 days respectively. The readmission rate in specialist paediatric centres was 6·3 per cent. Higher mortality was associated with birthweight lower than 1 kg (OR 5·90, 95 per cent c.i. 1·03 to 33·75), gestational age of 36 weeks or less (OR 1·75, 1·12 to 2·75) and black ethnicity (OR 2·13, 1·03 to 4·48). Only 4·0 per cent had extracorporeal membrane oxygenation, which was associated with higher mortality (OR 5·34, 3·01 to 9·46), longer nLOS (OR 3·70, 2·14 to 6·14) and longer total bed‐days (OR 3·87, 2·19 to 6·83). Specialist paediatric centres showed variation in 30‐day mortality (4·6 per cent with 84 per cent coefficient of variation), nLOS (median 25 (i.q.r. 15–42) days) and total bed‐days (median 28 (i.q.r. 16–51) days), but no significant volume–outcome relationship.

**Conclusion:**

Key outcomes for CDH were similar to those of other developed countries. High variation among specialist paediatric centres was found and should be investigated further to explore the value of regionalization of care.

## Introduction

Congenital diaphragmatic hernia (CDH) is a defect of the diaphragm causing part of the abdominal organs to herniate into the chest cavity. This can have a significant impact on lung development during fetal life and postnatal pulmonary hypertension, which is one of the main risk factors associated with death^1^. CDH has a prevalence rate of between 2·4 and 4·1 per 10 000 live births and affects approximately 200–300 infants in the UK annually^2^, ^3^. Despite advances in antenatal diagnosis and neonatal care, the overall mortality rate remains high, ranging from 42 to 68 per cent in population‐based studies^4^, ^5^.

Data from a perinatal confidential enquiry on CDH published in December 2014 showed large variations across the UK in terms of prenatal and postnatal management of women and babies diagnosed with CDH^6^. Many aspects of obstetric, neonatal and surgical care were shown to be without an adequate evidence base, leading to a recommendation to focus the acute care of babies with CDH to a limited number of specialist centres to improve quality of care, multidisciplinary work, audits and long‐term follow‐up.

There are no contemporary data on trends in incidence, variance in key outcomes, and the relationship between hospital volume and outcome among specialist paediatric surgery centres (SPCs) in England. A recent Canadian study^7^ found a positive relationship between hospital volume and survival in CDH. It is vital to have more data to confirm this association and support policy‐makers in deciding to regionalize care for this condition. In addition, to guide future service planning, reduce variation of care and the burden of this long‐term condition, it seemed essential to produce benchmark data for key 1‐year outcomes in England.

This study aimed to identify trends and factors associated with mortality, neonatal length of stay, readmission and total bed‐days within the first year of life for infants born with CDH between 2003 and 2016 in England. Variation in hospital outcomes and the existence of a relationship between hospital volume and outcome were analysed using data from SPCs.

## Methods

The Hospital Episode Statistics (HES) database records all completed consultant episodes for all admissions to National Health Service (NHS) hospitals in England. Approval from the Secretary of State and the Health Research Authority under Regulation 5 of the Health Service (Control of Patient Information) Regulations 2002 was gained to hold confidential data and analyse them for research purposes (Confidentiality Advisory Group; reference 15/CAG/0005), along with approval to use them for research and measuring quality of delivery of healthcare, from the London – South East Research Ethics Committee (REC reference 15/LO/0824).

The main problem treated (primary diagnosis), co‐morbidities and complications were coded using ICD‐10, and procedures were coded using the OPCS Classification of Interventions and Procedures (4th edition). Data on neonates younger than 28 days who had a primary diagnosis of CDH (Q79.0) between 1 April 2003 and 31 March 2016 were extracted from the HES database.

Procedure codes for open and thoracoscopic repair of CDH with and without a patch (*Table* *S1*, supporting information) were selected for this study. In addition, extracorporeal membrane oxygenation (ECMO) treatment was included as a marker of severity.

Twenty SPCs in England were identified to analyse variation in outcomes (*Table S2*, supporting information).

The main outcomes were 1‐year mortality, neonatal length of hospital stay (nLOS) (from birth to discharge from neonatal unit) and 1‐year total bed‐days (cumulative LOS within the first year of life). Variance among SPCs was studied for the main outcomes as well as 30‐day readmission and postoperative mortality. Volume–outcome analysis was conducted for patients who underwent repair in the 20 SPCs.

The following patient variables available within the HES database were included in the analysis: age at admission, sex (male or female), ethnicity (white, mixed, black, Asian, other ethnic group or missing), socioeconomic deprivation quintile (Carstairs deprivation index^8^), birthweight (999 g or less, 1000–2499 g and 2500 g or more), gestational weeks (26 weeks or less, 27–36 weeks and 37 weeks or more), year of admission, related procedures, and age at repair (days). Age at admission was the age that the baby was first recorded in the HES database, categorized by HES into three groups: 0, 1–6 and 6–28 days. Co‐morbidities known to be associated with CDH were included (ICD codes shown in *Table S3*, supporting information).

### Statistical analysis

Characteristics of mortality were compared with the χ^2^ test and length of stay/total bed‐days with the Kruskal–Wallis test. Factors associated with patient outcomes were identified using multivariable logistic regression models. All patients admitted to SPCs were included. Three distinct outcomes were modelled: mortality, nLOS and total bed‐days. nLOS and total bed‐days were categorized as ‘long’ or ‘normal’ based on their distribution for all patients admitted to SPCs. The 75th percentile was selected as the distinction between long and normal for both outcomes: for nLOS, long indicated more than 37 days and normal was 37 days or less; for total bed‐days, long indicated more than 45 days and normal was 45 days or less. Additionally, death within the first year of life was added in the models for predicting a longer nLOS and longer total bed‐days. Patients who died within the first year of life might have a shorter nLOS and fewer total bed‐days. All explanatory variables were retained in all models.

To investigate the association between SPC volume and outcomes, the total number of patients having surgical repair in each centre during the study period was calculated. Centres were divided into three groups based on tertiles: low volume, 60 patients or fewer who had CDH repair (232 patients, 7 centres); medium volume, 61–86 patients (525 patients, 7 centres); and high volume, 87 or more patients (627 patients, 6 centres). Initially, logistic regression with centre volume as the only explanatory variable was performed to obtain crude odds ratios (ORs). Multivariable logistic regression was used to calculate adjusted ORs in order to evaluate the effect of hospital volume on the three outcomes. Again, LOS and total bed‐days were converted into categorical variables using the 75th percentile to define long LOS and total bed‐days. The sample of patients was different to that in the main analysis and so the cut‐offs were different (42 days for LOS and 51 for total bed‐days). Sensitivity analyses were performed to assess the effect of different repair approaches by including the various approaches as predictors in the logistic regression model. All analyses were performed using SAS^®^ version 9.4 (SAS Institute, Cary, North Caroline, USA). Statistical significance was defined as *P* ≤ 0·050.

## Results

A total of 2379 patients were identified from the HES database as meeting the inclusion criteria from 2003 to 2016, of whom 2336 (98·2 per cent) were live births and 43 (1·8 per cent) were stillbirths. Of the live births, over half were diagnosed with one or more co‐morbidities; major cardiac malformations were the most common (38·7 per cent). Patient characteristics are summarized in *Table* 1.

**Table 1:** Summary of patient characteristics

	**Live births**	**1‐year mortality**	**nLOS (days)** *	**Total bed‐days** *, †
Total	2336	729 (31·2)	17 (3–33·5)	19 (5–40)
Age at admission (days)				
< 1	2074 (88·8)	692 (33·4)	17 (2–34)	19 (3–41)
1–6	173 (7·4)	31 (17·9)	15 (10–28)	17 (11–32)
7–28	89 (3·8)	6 (7)	14 (8–36)	17 (11–43)
*P*		0·178‡	< 0·001§	< 0·001§
Sex				
M	1369 (58·6)	402 (29·4)	17 (5–34)	19 (7–40)
F	954 (40·8)	323 (33·9)	16 (2–34)	19 (3–40)
n.s.	13 (0·6)	4 (31)	2 (0–2)	2 (0–2)
*P*		< 0·001‡	0·001§	< 0·001§
Birthweight (g)				
≥ 2500	1886 (80·7)	518 (27·5)	16 (5–30)	18 (7–35)
1000–2499	412 (17·6)	184 (44·7)	22 (1–55)	25 (2–70)
≤ 999	38 (1·6)	27 (71)	3·5 (0–53)	3 (0–54)
*P*		0·001‡	0·004§	0·001§
Gestational week				
≥ 37	2024 (86·6)	594 (29·3)	16 (4–31)	18 (6–36)
27–36	289 (12·4)	117 (40·5)	26·5 (1–54)	29 (1–71)
≤ 26	23 (1·0)	18 (78)	5 (0–39)	5 (0–67)
*P*		< 0·001‡	< 0·001§	< 0·001§
No. of co‐morbidities				
0	1110 (47·5)	297 (26·8)	13 (2–15)	15 (2–28)
≥ 1	1226 (52·5)	432 (35·2)	22 (6–45)	25 (8–54)
*P*		< 0·001	< 0·001§	< 0·001§
Ethnicity				
White	1575 (67·4)	419 (26·6)	18 (7–35)	20 (9–43)
Mixed	64 (2·7)	20 (31·3)	22·5 (2·0–31·5)	23·5 (3·5–32·5)
Asian	323 (13·8)	85 (26·3)	21 (7–37)	23 (11–45)
Black	85 (3·6)	36 (42)	15 (2–43)	21 (2–50)
Other	67 (2·9)	26 (39)	19 (2–30)	20 (2–34)
Missing	222 (9·5)	143 (64·4)	2 (1–14)	2 (1–14)
*P*		< 0·001‡	< 0·001§	< 0·001§
Socioeconomic quintile				
1 (least deprived)	294 (12·6)	37 (12·6)	21 (12–38)	23 (13–45)
2	263 (11·3)	46 (17·5)	19 (11–35)	23 (13–48)
3	348 (14·9)	66 (19·0)	22 (11–37)	25 (13·5–49)
4	424 (18·2)	99 (23·3)	22 (11·5–40·5)	25·5 (13–49)
5 (most deprived)	499 (21·4)	108 (21·6)	22 (11–42)	25 (13–50)
6 (missing)	508 (21·7)	373 (73·4)	1 (1–4)	1 (0–4)
*P*		< 0·001‡	< 0·001§	< 0·001§

Values in parentheses are percentages unless indicated otherwise;

* values are median (i.q.r.).

† Cumulative 1‐year total bed‐days. nLOS, neonatal length of hospital stay; n.s. not specified.

‡ χ^2^ test;

§ Kruskal–Wallis test.

The overall prevalence of CDH in England, based on the cohort, was estimated to be 2·5 per 10 000 live births. No significant time trend in incidence was observed over the study interval (*P* = 0·085). Although mortality, nLOS and total bed‐days fluctuated by year, there was no significant trend in these three outcomes over the years. Crude associations between patient characteristics and 1‐year mortality, nLOS and total bed‐days are shown in *Table* 1. The majority of patient characteristics were significantly associated with worse outcomes. Being male was associated with longer nLOS and lower mortality compared with being female. Socioeconomic deprivation and the presence of one or more co‐morbidities were associated with poorer outcomes.

Among 2336 live births, 1491 (63·8 per cent) underwent surgical repair in the first few months of life; 25·9 per cent died within the first year of life without undergoing repair. The most common operation to repair CDH was an open repair without a patch (1387 patients, 93·0 per cent). Repairs in the other 104 patients included: unspecified other approach (89), thoracoscopic repair (9) and open repair with a patch (1) (data were missing for the other 5 patients). The numbers of patients receiving patch repair or a minimally invasive procedure were extremely low, although this may reflect poor coding quality related to the type of surgery used. Some 4·0 per cent of total live births were recorded as having received ECMO (*Table* 2).

**Table 2:** Summary of repairs for all live births

	**No. of live births (*n* = 2336)**
Death before repair	604 (25·9)*
Primary repair	1491 (63·8)*
Open repair without patch	1387 (93·0)†
Other approach	104 (7·0)†
Redo operation	102 (6·8)†
Death after repair	125 (8·4)†
Extracorporeal membrane oxygenation	94 (4·0)*

Values in parentheses are percentages of

* all live births and

† patients who had a repair.

### Factors associated with patient outcomes

Data for patients admitted to SPCs were included in multivariable logistic models. The results are summarized in *Table* 3.

**Table 3:** Multivariable analysis of factors predicting 1‐year mortality, neonatal length of stay longer than 37 days, and total bed‐days longer than 42 days in patients with congenital diaphragmatic hernia

	**1‐year mortality**		**nLOS > 37 days**		**Total bed‐days > 42 days**	
	**Odds ratio**	***P***	**Odds ratio**	***P***	**Odds ratio**	***P***
Age at admission (days)						
0	1·00 (reference)		1·00 (reference)		1·00 (reference)	
1–6	1·05 (0·59, 1·87)	0·873	0·60 (0·37, 0·96)	0·035	0·57 (0·35, 0·94)	0·027
7–28	0·07 (0·02, 0·27)	< 0·001	0·90 (0·48, 1·70)	0·752	0·90 (0·47, 1·73)	0·755
Sex						
M	1·00 (reference)		1·00 (reference)		1·00 (reference)	
F	0·92 (0·68, 1·25)	0·605	0·94 (0·73, 1·21)	0·624	1·15 (0·89, 1·49)	0·286
Birthweight (g)						
≥ 2500	1·00 (reference)		1·00 (reference)		1·00 (reference)	
1000–2499	2·37 (1·60, 3·50)	< 0·001	2·91 (2·11, 4·03)	< 0·001	3·07 (2·20, 4·30)	< 0·001
≤ 999	5·90 (1·03, 33·75)	0·046	6·89 (1·74, 27·27)	0·006	4·18 (0·96, 18·23)	0·057
Gestational weeks						
≥ 37	1·00 (reference)		1·00 (reference)		1·00 (reference)	
27–36	1·75 (1·12, 2·75)	0·015	2·20 (1·52, 3·18)	< 0·001	2·51 (1·72, 3·67)	< 0·001
≤ 26	5·73 (0·87, 37·72)	0·070	0·55 (0·09, 3·50)	0·524	2·99 (0·56, 15·94)	0·199
Ethnicity						
White	1·00 (reference)		1·00 (reference)		1·00 (reference)	
Mixed	1·13 (0·47, 2·69)	0·784	0·99 (0·46, 2·12)	0·981	0·94 (0·42, 2·10)	0·874
Asian	0·94 (0·58, 1·51)	0·789	1·32 (0·92, 1·91)	0·132	1·36 (0·93, 1·98)	0·114
Black	2·13 (1·03, 4·48)	0·046	2·36 (1·20, 4·61)	0·012	2·30 (1·16, 4·57)	0·017
Chinese	0·67 (0·26, 1·71)	0·403	1·10 (0·52, 2·32)	0·811	0·82 (0·36, 1·86)	0·635
Missing	3·25 (1·82, 5·80)	< 0·001	0·70 (0·39, 1·26)	0·237	0·73 (0·40, 1·34)	0·307
Socioeconomic quintile						
1 (least deprived)	1·00 (reference)		1·00 (reference)		1·00 (reference)	
2	1·37 (0·73, 2·59)	0·327	0·92 (0·59, 1·45)	0·723	1·20 (0·75, 1·90)	0·447
3	1·59 (0·88, 2·88)	0·123	0·93 (0·61, 1·42)	0·744	1·12 (0·73, 1·73)	0·598
4	1·96 (1·11, 3·46)	0·021	1·12 (0·75, 1·69)	0·573	1·10 (0·72, 1·69)	0·649
5 (most deprived)	1·86 (1·06, 3·28)	0·032	1·13 (0·75, 1·68)	0·565	1·27 (0·84, 1·93)	0·260
Missing	7·43 (4·05, 13·63)	< 0·001	0·56 (0·31, 1·01)	0·054	0·45 (0·24, 0·86)	0·015
Co‐morbidity						
None	1·00 (reference)		1·00 (reference)		1·00 (reference)	
Major cardiac anomaly	1·15 (0·83, 1·60)	0·406	1·52 (1·18, 1·97)	0·001	1·71 (1·32, 2·23)	< 0·001
Primary pulmonary hypertension	2·30 (1·44, 3·65)	< 0·001	2·87 (1·94, 4·24)	< 0·001	2·85 (1·90, 4·26)	< 0·001
Congenital hypoplasia of lung	2·79 (1·97, 3·96)	< 0·001	2·85 (2·11, 3·86)	< 0·001	3·19 (2·34, 4·34)	< 0·001
Chronic lung disease	0·21 (0·07, 0·68)	0·009	11·83 (4·39, 31·85)	< 0·001	22·16 (6·47, 75·86)	< 0·001
Secondary pulmonary hypertension	0·36 (0·12, 1·09)	0·070	3·84 (1·73, 8·52)	0·001	6·08 (2·56, 14·44)	< 0·001
Chromosomal abnormalities	2·26 (1·03, 4·98)	0·044	2·05 (1·03, 4·09)	0·042	2·95 (1·47, 5·93)	0·002
Surgery						
Open repair	1·00 (reference)		1·00 (reference)		1·00 (reference)	
Thoracoscopic repair	0·32 (0·04, 2·44)	0·271	0·23 (0·08, 0·69)	0·009	0·31 (0·11, 0·87)	0·026
ECMO	5·34 (3·01, 9·46)	< 0·001	3·70 (2·14, 6·41)	< 0·001	3·87 (2·19, 6·83)	< 0·001
Death within 365 days	–	–	0·29 (0·19, 0·43)	< 0·001	0·25 (0·16, 0·38)	< 0·001

Values in parentheses are 95 per cent confidence intervals. nLOS, neonatal length of hospital stay; ECMO, extracorporeal membrane oxygenation.

Older age at admission and chronic lung disease were associated with lower mortality. Low birthweight (999 g or less) was associated with a nearly sixfold higher odds of mortality, as was gestational age of 26 weeks or less, in comparison with birthweight of 2500 g or more and full‐term respectively. Babies of black ethnicity had twice the odds of mortality as babies of white ethnicity (OR 2·13, 95 per cent c.i. 1·03 to 4·48). Primary pulmonary hypertension, congenital hypoplasia of lung and chromosomal abnormalities were all associated with increased mortality.

Multivariable logistic analysis of longer nLOS suggested that low birthweight, preterm delivery, black ethnic group, presence of major cardiac anomalies, primary pulmonary hypertension, congenital hypoplasia of lung, chronic lung disease, chromosomal abnormalities and secondary pulmonary hypertension were risk factors for a longer LOS. Factors independently associated with a shorter LOS included older age at admission, death within the first year of life, and receiving thoracoscopic repair. Similar results were observed in the logistic regression model for predicting longer total bed‐days.

The receipt of ECMO, included as a marker of severity, showed that babies receiving this treatment had an increased risk of death (OR 5·34, 95 per cent c.i. 3·01 to 9·46), longer nLOS (OR 3·70, 2·14 to 6·14) and longer total bed‐days (OR 3·87, 2·19 to 6·83).

### Variation between specialist paediatric centres

Some 1384 patients underwent repair in specialist centres, and only 107 were recorded as having their repair in a non‐specialist centre (*Table* 4).

**Table 4:** Comparison of outcomes for neonates who underwent repair among specialist paediatric centres

	**No. repaired**	**Age at operation (days)** *	**nLOS (days)** *	**Total bed‐days (days)** *	**30‐day readmission rate (%)**	**30‐day postoperative mortality rate (%)**
Specialist centres	1384	4·0	25·0	28·0	6·3	4·6
A	86	5·0	23·5	29·5	7	1
B	92	3·0	22·0	25·0	5	5
C	58	3·5	28·0	32·0	5	4
D	60	3·0	20·0	23·0	5	7
E	80	2·0	21·5	25·0	6	0
F	20	3·0	21·0	27·5	0	6
G	64	4·0	24·5	27	8	7
H	101	3·0	33·0	36	2·0	8·7
I	27	2·0	25·0	28	0	0
J	126	7·0	26·5	33	12·7	4·8
K	103	7·0	29·0	36	4·9	1·1
L	68	5·0	28·5	30·5	7	2
M	78	3·0	24·5	29·5	6	3
N	88	3·0	27·5	29·5	9	5
O	67	2·0	18·0	20	12	2
P	117	4·0	26·0	28	5·1	1·9
Q	3	3·5	21·0	27	0	0
R	82	3·0	21·0	23·5	5	16
S	54	3·0	18·0	21	2	4
T	10	3·0	34·5	34·5	0	0
Other centre	107	4·0	21·0	25·0	6·5	0·0
Total	1491	4·0	25·0	28	6·3	4·0

* Values are median. nLOS, neonatal length of hospital stay.

**Figure 1:**
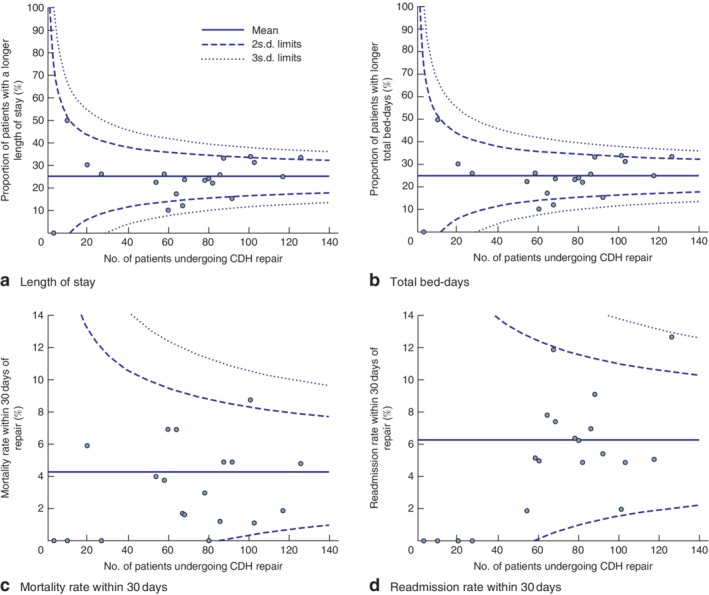
Funnel plots for main outcomes in patients undergoing repair of congenital diaphragmatic hernia in specialist paediatric centres in England, 2003–2016. **a** Length of hospital stay, **b** total bed‐days, **c** mortality within 30 days and **d** readmission within 30 days for 20 specialist paediatric centres. CDH, congenital diaphragmatic hernia.

Median nLOS was 25 (i.q.r. 15–42) days, indicating a significant degree of variation among the SPCs. Median total bed‐days was 28 (i.q.r. 16–51) days. In SPCs the mortality rate within 30 days of the index repair was 4·6 per cent with 84 per cent coefficient of variation. Variation was observed between centres (standard deviation 3·86 for mortality and 3·64 for readmission). The coefficient of variation was high for both mortality and readmission (99 and 71 per cent respectively). *Fig*. 1 presents funnel plots for the main outcomes in patients having repair in SPCs. The data suggest that the majority of variation in mortality and readmission rates within 30 days between centres was within that expected by chance alone. For mortality, two centres were outside the 95 per cent control limits. For readmission, a different centre was outside the 95 per cent limits.

### Volume–outcome relationship in specialist paediatric centres

The volume–outcome relationship is summarized in *Table* 5. When crude outcomes were considered there was some evidence of a volume–outcome relationship. There was a higher mortality rate in patients who had repair in units that performed a higher volume of repairs. A similar pattern was observed for LOS and total bed‐days. However, for all key outcomes, when adjusted for patient's age at admission, sex, ethnicity, quintile, birthweight, gestational weeks, co‐morbidities and ECMO, there were no significant differences in odds of outcomes for patients treated in high‐, medium‐ or low‐volume centres. Sensitivity analysis indicated that there was still no significant volume–outcome relationship after including procedure attributes in the logistic regression models.

**Table 5:** 1‐year mortality rate, 30‐day readmission rate, postoperative length of stay, total bed‐days and odds ratios by hospital repair volume groups

	**Hospital volume**		
	**≤ 60**	**61–86**	**≥ 87**
1‐year mortality rate (%)	5·2	7·8	10·5
Crude OR	1·00 (reference)	1·55 (0·80, 3·01)	2·16 (1·14, 4·07)
Adjusted OR†	1·00 (reference)	1·36 (0·67, 2·77)	1·77 (0·89, 3·53)
Readmission rate (%)	57·3	49·3	58·7
Crude OR	1·00 (reference)	0·73 (0·53, 0·99)	1·06 (0·78, 1·44)
Adjusted OR†	1·00 (reference)	0·82 (0·59, 1·14)	0·99 (0·71, 1·37)
Postoperative LOS (days)*	23 (13–38)	23 (14–38)	27 (16–49)
Crude OR	1·00 (reference)	1·03 (0·79, 1·34)	1·46 (1·12, 1·89)
Adjusted OR†	1·00 (reference)	0·83 (0·54, 1·28)	1·04 (0·69, 1·57)
Total bed‐days*	25 (15–48)	27 (16–46)	30 (18–56)
Crude OR	1·00 (reference)	1·15 (0·88, 1·50)	1·54 (1·19, 2·00)
Adjusted OR†	1·00 (reference)	0·83 (0·53, 1·29)	0·95 (0·62, 1·46)

Values in parentheses are 95 per cent confidence intervals unless indicated otherwise;

* values are median (i.q.r.). OR, odds ratio; LOS, length of hospital stay.

† Adjusted for patient age at admission, sex, ethnicity, socioeconomic quintile, birthweight, gestational age, presence of co‐morbidities and extracorporeal membrane oxygenation.

## Discussion

This study has evaluated trends and outcomes associated with CDH treated in England over a 13‐year period. The overall 1‐year mortality rate was 31·2 per cent. There was no significant time trend in incidence, crude rates of mortality, nLOS or total bed‐days. Prematurity below 37 weeks of gestation and low birthweight were significantly associated with higher mortality and LOS. Babies born with a birthweight below 1 kg had sixfold higher odds of death than those with a birthweight of 2·5 kg or more. Independent patient‐level risk factors associated with CDH mortality and longer nLOS and total bed‐days were: primary pulmonary hypertension, congenital hypoplasia of lung and chromosomal abnormalities. The occurrence of chronic lung disease was associated with a decreased risk of 1‐year mortality; this is explained by the fact that only infants who survive to 28 days can be diagnosed with chronic lung disease. Receipt of ECMO, which was considered a marker of severity, was associated with poorer outcomes. There was variation in outcomes among SPCs. Unadjusted ORs suggested that higher‐volume centres had poorer outcomes, but when these were adjusted for patient characteristics and procedures there was no evidence of a volume–outcome relationship.

In the last decade in England, the mortality rate from CDH (31·2 per cent) has continued to decline compared with historical rates, reported as 50 per cent between 1985 and 1997, and 39 per cent between 1997 and 2005^9^. A study^10^ conducted in Sweden reported a significant reduction in mortality between two historical cohorts (1995–2005 and 2006–2016), from 17·9 to 4·4 per cent. This improvement was explained by regionalization of care and *ad hoc* treatment protocols in neonatal ICUs. A study^11^ based on a single American institution reported an overall mortality rate of 19 per cent, with no difference in mortality across four study eras from 1998 to 2003, although there was an improvement in survival after the introduction of ECMO. An improved survival rate using ECMO was not shown in the present cohort, possibly reflecting a higher threshold of severity needed before ECMO is used in England compared with that in America.

No significant relationship was found in the present study between 1‐year mortality and the presence of major cardiac anomalies in CDH after risk adjustments. Conversely, other studies^12^, ^13^ have reported a mortality rate of up to 60 per cent in patients with associated major cardiac anomalies.

Although there was no significant trend in LOS over time, a single‐institution study^11^ reported that LOS increased over the years, perhaps indicating that, in more recent years, sicker babies have become more likely to survive with a need for a longer hospital stay.

A similar result to that found here was reported in an American study^14^, in which the mortality rate was found to be higher in patients with black ethnicity than in white and Asian ethnic groups. The higher 1‐year mortality rate found in black babies compared with other ethnic groups may reflect poorer access to antenatal care. A previous study^15^ demonstrated that black women in England were more likely to book their antenatal healthcare appointment after 18 weeks of gestation against the international recommendation to start antenatal care within the first trimester. In this study, ethnicity may be acting as a proxy for measures of deprivation not included in the composite measure.

A relationship between hospital volume and outcome studied among the SPCs was not apparent in the present study. This result is consistent with a large retrospective cohort study^16^ from academic medical centres in the USA, which showed no difference in mortality between low‐ and high‐volume centres. Conversely, two other studies^7^, ^17^ found a significant volume–outcome relationship in CDH, although their definitions of a high‐volume hospital were more than six and more than ten repairs a year, which would include all but one of the SPCs in England.

Based on these results, policy‐makers, hospitals, doctors, parents and CDH associations will have solid data to reduce medical and surgical variation of care. Internationally validated protocols for prenatal and postnatal management of this condition are needed to reduce variation of care and to compare outcomes objectively. Systematic monitoring of outlier hospitals dealing with CDH will drive future regionalization of care to focus resources and enhance quality of care for this complex multidisciplinary condition.

This study did not show any reduction in mortality in the past decade. This is likely to be related to the small number of fetuses that received prenatal treatment in England. Results from specialist centres also did not support centralization of care for CDH. As demonstrated for other neonatal conditions, however, high‐volume hospitals may provide more holistic and coordinated care for babies diagnosed with CDH *in utero*. This study does support the need for more research to evaluate cost‐effectiveness and composite outcomes in an attempt to define benefits of regionalization of care. In particular, maternal health records, termination rate and spontaneous abortion rate should be included to obtain the incidence and mortality in these fetuses. Further research is needed to understand whether prenatal treatments (fetal tracheal occlusion^18^ and novel prenatal drugs treating pulmonary hypertension) will be able to improve mortality and 1‐year outcomes. In terms of postnatal treatment, the indication for the use of ECMO should also be standardized internationally to facilitate comparisons and develop clinical trials investigating effectiveness.

This study was not able to include several important indicators of CDH severity. Although ECMO was included as a marker, the low prevalence of ECMO (4·0 per cent) suggests that this procedure is not widely available in England compared with American hospitals, where up to 22 per cent of babies with CDH receive it^19^. Apgar score, side and size of the defect, and surgical technique used for the repair (open *versus* thoracoscopic) are commonly included in similar studies as indicators of disease severity. Unfortunately these data were either not available or not recorded accurately in the HES data set. Previous studies also included the use of patch repair for diaphragmatic repair as a robust surrogate marker of disease severity. Owing to the low prevalence of patch repair, which might be attributed to coding error, this variable could also not be included in the regression model. According to previous studies, the diagnostic accuracy was found to be 96·0 (i.q.r. 89·3–96·3) per cent from 2002 onwards, whereas the accuracy of procedure coding ranged from 83·4 to 87·0 per cent^20^. Additionally, a large proportion of stillbirths were not recorded in the HES births data set, which might have caused underestimation of incidence and mortality. In addition, this study attributed the outcomes to centres with CDH recorded first, and did not consider transfers of patients between centres as contributing to patient outcomes. Missing deprivation and ethnicity data (21·7 and 9·5 per cent respectively) may also have impacted on some results.

## Supporting information


**Table S1** OPCS‐4 procedure codes for the treatment of congenital diaphragmatic hernia
**Table S2** Hospital codes for specialist paediatric centres in England
**Table S3** Co‐morbidities and ICD‐10 codes
