# Supplementary material for: One‐year outcomes for congenital diaphragmatic hernia
Source: BJS Open. 2019 Jan 31;3(3):305–13. doi: 10.1002/bjs5.50135 (PMC6551417; doi:10.1002/bjs5.50135)
Supplement: Supplementary file 1 — Table S1 OPCS‐4 procedure codes for the treatment of congenital diaphragmatic hernia Table S2 Hospital codes for specialist paediatric centres in England Table S3 Co‐morbidities and ICD‐10 codes [file BJS5-3-305-s001.docx]

**BJS5_50135**

**One-year outcomes for congenital diaphragmatic hernia**

**Y. Wang, K. Honeyford, P. Aylin, A. Bottle and S. Giuliani**

**Table S1 OPCS-4 procedure codes for the treatment of congenital diaphragmatic hernia**

| **Description** | | **OPCS-4 code** |
| --- | --- | --- |
| Open repair with sutures | Repair of congenital diaphragmatic hernia | T164 |
|  | Repair of diaphragmatic hernia using abdominal approach NEC | G234 |
|  | Plication of diaphragm | T162 |
| Thoracoscopic repair with sutures | Thoracoscopic approach to thoracic cavity NEC | Y742 |
|  | Repair of diaphragmatic hernia using thoracic approach NEC | G232 |
| Patch repair | Insertion of prosthesis for repair of diaphragm | T161 |
|  | Unspecified other repair of diaphragm | T169 |
|  | Unspecified repair of diaphragmatic hernia | G239 |
| ECMO | Extracorporeal membrane oxygentaion | X58.1 |

Table S2 Hospital codes for specialist paediatric centres in England

| **Code** | **Hospital Name** |
| --- | --- |
| RA7 | UNIVERSITY HOSPITALS BRISTOL NHS FOUNDATION TRUST |
| RBS | ALDER HEY CHILDREN'S NHS FOUNDATION TRUST |
| RCU | SHEFFIELD CHILDREN'S NHS FOUNDATION TRUST |
| RGT | CAMBRIDGE UNIVERSITY HOSPITALS NHS FOUNDATION TRUST |
| RHM | UNIVERSITY HOSPITAL SOUTHAMPTON NHS FOUNDATION TRUST |
| RJ1 | GUY'S AND ST THOMAS' NHS FOUNDATION TRUST |
| RJ7 | ST GEORGE'S HEALTHCARE NHS TRUST |
| RJZ | KING'S COLLEGE HOSPITAL NHS FOUNDATION TRUST |
| RM1 | NORFOLK AND NORWICH UNIVERSITY HOSPITALS NHS FOUNDATION TRUST |
| RP4 | GREAT ORMOND STREET HOSPITAL FOR CHILDREN NHS FOUNDATION TRUST |
| RQ3 | BIRMINGHAM CHILDREN'S HOSPITAL NHS FOUNDATION TRUST |
| RQM | CHELSEA AND WESTMINSTER HOSPITAL NHS FOUNDATION TRUST |
| RR8 | LEEDS TEACHING HOSPITALS NHS TRUST |
| RTD | THE NEWCASTLE UPON TYNE HOSPITALS NHS FOUNDATION TRUST |
| RTH | OXFORD UNIVERSITY HOSPITALS NHS TRUST |
| RW3 | CENTRAL MANCHESTER UNIVERSITY HOSPITALS NHS FOUNDATION TRUST |
| RWA | HULL AND EAST YORKSHIRE HOSPITALS NHS TRUST |
| RWE | UNIVERSITY HOSPITALS OF LEICESTER NHS TRUST |
| RX1 | NOTTINGHAM UNIVERSITY HOSPITALS NHS TRUST |
| RXH | BRIGHTON AND SUSSEX UNIVERSITY HOSPITALS NHS TRUST |

**Table S3 Co-morbidities and ICD-10 codes**

| **ICD-10 code** | **Description** |
| --- | --- |
| Q20-26 | Major cardiac anomalies |
| Q89.7 | Multiple congenital anomalies |
| I27.0 | Primary Pulmonary Hypertension |
| I27.2 | Secondary PHP |
| Q90-93, Q96-Q99, excludes Q93.6, Q95 | Chromosomal abnormalities |
| Q33.6 | Congenital hypoplasia of lung |
| P27.9 | Chronic lung disease infant |
